# Antibody Response to SARS-CoV-2 Vaccination in Heart Failure Patients: Retrospective Single-Center Cohort Study

**DOI:** 10.3390/diagnostics13223460

**Published:** 2023-11-16

**Authors:** Defne Güneş Ergi, Ümit Kahraman, Gözde Akkuş, Seyfi Durmaz, Özlem Balcıoğlu, Çağatay Engin, Burcu Yağmur, Sanem Nalbantgil, Candan Çiçek, Mustafa Özbaran, Tahir Yağdı

**Affiliations:** 1 Department of Cardiovascular Surgery, Faculty of Medicine, Ege University, Bornova, 35100 Izmir, Turkey; gunes_ergi@hotmail.com (D.G.E.); umitkahraman81@gmail.com (Ü.K.); cagatayengin@yahoo.com (Ç.E.); mustafa.ozbaran@gmail.com (M.Ö.); 2 Department of Microbiology, Faculty of Medicine, Ege University, Bornova, 35100 Izmir, Turkey; gozdeakkus1306@hotmail.com (G.A.); candan.cicek@ege.edu.tr (C.Ç.); 3 Department of Public Health, Faculty of Medicine, Ege University, Bornova, 35100 Izmir, Turkey; seyfidurmaz@gmail.com; 4 Department of Cardiovascular Surgery, Near East University Hospital, 99138 Nicosia, Cyprus; ozlem.balcioglu@neu.edu.tr; 5 Department of Cardiology, Faculty of Medicine, Ege University, Bornova, 35100 Izmir, Turkey; burcuyagmur121@gmail.com (B.Y.); sanemn@hotmail.com (S.N.)

**Keywords:** heart failure, left ventricular assist device, vaccination, immunity, infection

## Abstract

We sought to investigate the impact of heart failure on anti-spike antibody positivity following SARS-CoV-2 vaccination. Our study included 103 heart failure (HF) patients, including those with and without left ventricular assist devices (LVAD) selected from our institutional transplant waiting list as well as 104 non-heart failure (NHF) patients who underwent open heart surgery at our institution from 2021 to 2022. All the patients received either heterologous or homologous doses of BNT162b2 and CoronaVac. The median age of the HF group was 56.0 (interquartile range (IQR): 48.0–62.5) and the NHF group was 63.0 (IQR: 56.0–70.2) years, and the majority were males in both groups (*n* = 78; 75.7% and *n* = 80; 76.9%, respectively). The majority of the patients in both the HF and NHF groups received heterologous vaccinations (*n* = 43; 41.7% and *n* = 52; 50.3%, respectively; *p* = 0.002). There was no difference in the anti-spike antibody positivity between the patients with and without heart failure (*p* = 0.725). Vaccination with BNT162b2 led to significantly higher antibody levels compared to CoronaVac alone (OR: 11.0; 95% CI: 3.8–31.5). With each passing day after the last vaccine dose, there was a significant decrease in anti-spike antibody positivity, with an OR of 0.9 (95% CI: 0.9–0.9). Furthermore, hyperlipidemia was associated with increased antibody positivity (*p* = 0.004).

## 1. Introduction

In 2019, the global population faced a threat in the form of an emerging pandemic caused by the severe acute respiratory syndrome coronavirus 2 (SARS-CoV-2). The pandemic led to extensive worldwide efforts to develop immunity against the virus, resulting in the creation of multiple COVID-19 vaccines that played a vital role in curbing hospitalizations, preventing admissions to intensive care units, and reducing the number of fatalities. These vaccines encompassed various types, including inactivated whole virus vaccines, lipid-nanoparticle-encapsulated mRNA vaccines, adenovirus-vector vaccines, and protein subunit vaccines [1].

The Pfizer COVID-19 vaccine, which was designated as BNT162b2, was formulated using lipid nanoparticles containing modified RNA targeting the spike protein of the SARS-CoV-2 virus. Studies have indicated an impressive 95% effectiveness in safeguarding against COVID-19 when the vaccine was administered in a two-dose regimen [2]. Another significant vaccine is CoronaVac (Sinovac Life Sciences in Beijing, China), which was constructed using an inactivated whole virus approach with an aluminum-hydroxide adjuvant. This vaccine was developed using African green monkey kidney cells that were exposed to the SARS-CoV-2 virus. The efficacy and safety of the CoronaVac were also granted approval [3,4].

In individuals with advanced heart failure, the immune system becomes activated within the context of heart failure. This activation is accompanied by heightened levels of certain molecules and compounds, such as the membrane attack complex (formed owing to complement activation), interleukin-6, tumor necrosis factor-α, and interleukin-1 [5,6]. Furthermore, patients who receive a circulatory support from a left ventricular assist device (LVAD) often encounter infection-related complications. Interestingly, this immune dysregulation can initiate even before LVAD implantation. Evidence indicates that activated monocytes (a type of immune cell) and T-cells (another type of immune cell) accumulate on the surface of LVADs. These cells are prone to undergoing apoptosis (cell death) upon further stimulation. This process leads to a state of relative T-cell-focused immune suppression. In essence, the immune response becomes skewed toward suppressing T-cell activity, which can have implications for the overall immune competence of these patients [5,6]. 

In addition to the immune-related effects of heart failure and LVADs, other factors contribute to immune system alterations in these patient groups. For instance, advanced age and diabetes can further impact the functionality of the immune system [7]. The immune system’s ability to respond to challenges, ward off infections, and maintain a balanced state can be compromised due to these complex interactions. 

The primary objective of the current study was to analyze and compare the variations in antibody positivity triggered by vaccination against SARS-CoV-2 in two distinct groups: individuals with heart failure and individuals without heart failure. Additionally, we aimed to investigate the variations in anti-spike antibody levels based on the specific type of vaccination and how these levels changed over time following the last vaccine dose. By addressing these questions, the present study aimed to contribute to the understanding of how heart failure impacts the immune response to SARS-CoV-2 vaccinations and whether certain vaccine types might be more effective in generating a robust antibody response.

## 2. Materials and Methods

As a part of our transplant program, we are overseeing the care of 155 heart failure (HF) patients with a left ventricular assist device (LVAD) along with 47 patients without an LVAD. We identified 202 HF patients (both with and without an LVAD) from our institutional ‘heart transplant waiting list’ to be included in the study. For comparison, we identified another group of 182 non-heart failure (NHF) patients who had undergone open heart surgery under the care of our transplant team from January 2021 through January 2022. The NHF group had normal ventricular dimensions, normal ejection fractions, and no signs or symptoms of heart failure. 

**Figure 1:**
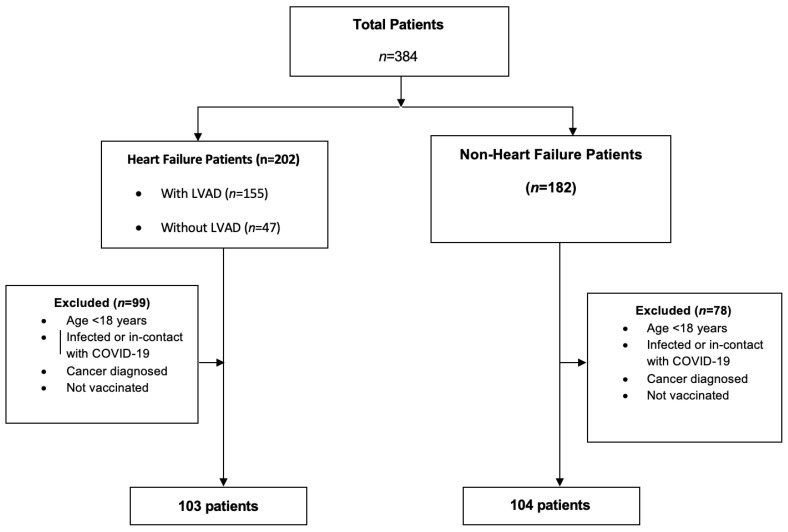
Flowchart of the study.

Patients were included in the study if they had received at least the primary series of SARS-CoV-2 vaccinations, which was defined as the receipt of two vaccine doses for persons who had received Pfizer-BioNTech, MODERNA mRNA-1273 vaccine (Cambridge, MA, USA), or unspecified U.S.-authorized or -approved mRNA COVID-19 vaccines [8]. Exclusion criteria included being under 18 years of age, having a history of cancer, and not being immunized. As a result, 78 patients in the NHF group and 99 in the HF group were excluded (Figure 1). The final study cohort consisted of 207 patients.

It is worth noting that warfarin therapy following valve-replacement surgery and inflammation induced by open heart surgery were not considered as immunocompromised statuses. The relevant demographic data, baseline comorbidities, and details of the surgical procedure were obtained through a review of electronic medical records. This study was approved by the Ege University Faculty of Medicine Institutional Review Board (Approval: 22-3.3/5) and this work was supported by the Ege University Scientific Research Projects Coordination Unit (Project Number: 23650). All the patients provided their consent for participation.

### 2.1. Data Collection and Measures

An experienced healthcare professional obtained one tube of venous blood from each participant, which amounted to approximately twenty milliliters, during their routine follow-up visit to the outpatient clinic. The blood samples were collected in blood collection tubes (VACUSERA 2 mL Serum Gel and Clot Activator) for plasma separation. The first collected sample was stored at +4 °C in the refrigerator for one day. Then, the samples were centrifuged at 2000 g for 20–30 min for the plasma to be stored until testing. An enzyme-linked immunosorbent assay (EUROIMMUN Anti-SARS-CoV-2 QuantiVac IgG ELISA) was used for the quantitative in vitro determination of human anti-spike antibodies of immunoglobulin class IgG against the SARS-CoV-2 spike protein in the EUROIMMUNE analyzer (Medizinische Labordiagnostika AG, Lübeck, Germany). The sample collection date of each participant was recorded and calculated according to the time interval from the last dose of the vaccine.

The primary outcome of the study was set at anti-spike antibody positivity. Because there were no established international quantitative reference values for specific antibodies against SARS-CoV-2. The equipment calibration was assessed in Relative Units (RUs). This ratio was interpreted as follows: <8 RU/mL: negative; from ≥8 RU/mL to <11 RU/mL: borderline; ≥11 RU/mL: positive. Borderline results were considered as negative for the analysis [9,10].

### 2.2. Statistical Analysis

Categorical variables are presented as numbers (percentages), while continuous variables are expressed as medians with interquartile ranges (25th–75th percentiles). Group comparisons utilized either the Mann–Whitney U test (the normality was checked according to the Kolmogorov–Smirnov test) or Pearson’s Chi-Squared test, depending on appropriateness. When assessing antibody levels, values exceeding 120 (37.7%) and those below 1 (2.4%) were unmeasurable. Consequently, instead of numerical assessment, antibody positivity was determined based on a predefined cutoff point (≥11 RU/mL). However, the relationship between the numerical antibody levels and time elapsed since the last vaccine dose during sample collection was given graphically separately in groups according to vaccine types. Owing to variations in the vaccine combinations and time elapsed since the last vaccine dose during sample collection, the antibody evaluation lacked independence from these factors. As a result, antibody positivity was assessed in regression approach instead of univariate analysis. In logistic regression, potential confounders, excluding the duration and vaccine type, were considered based on their clinical significance. Variables, such as age and gender; hypertension, diabetes, hyperlipidemia, and vaccination statuses; the time interval between the vaccination and blood sample collection; HF versus NHF; and the interaction between the time and HF versus time and NHF, were included in the multivariable logistic regression to predict anti-spike antibody positivity. Model outcomes were reported as odds ratios (ORs) with corresponding 95% confidence intervals (CIs). The forward stepwise elimination method was employed in the multivariable logistic regression. As HF versus NHF could not be included in the final model owing to forward elimination and considering the main study hypothesis centered around the HF versus NHF comparison, both group and group-time interactions were added to the final model selected in forward elimination. The results for this second model, testing the time effect on antibody positivity across the groups, are presented separately. The level of significance was set at *p* < 0.05. All the statistical analyses were performed using SPSS software version 22.0 (IBM, Armonk, NY, USA).

## 3. Results

### 3.1. Baseline Characteristics

The median age was 56.0 years (IQR, 48.0–62.5) in HF group and 63.0 years (IQR, 56.0–70.2) in NHF group. The majority were males in both groups (*n* = 78; 75.7% and *n* = 80; 76.9%, respectively). Among the NHF group, the most common cardiac operation was coronary artery bypass grafting (*n* = 65, 62.5%). Isolated aortic valve replacement was the second most common procedure (*n* = 10, 9.6%). The majority of the HF patients had dilated cardiomyopathy (*n* = 50, 48.5%). Among the HF group with an LVAD, the majority had a HeartWare HVAD (Medtronic) device (*n* = 62, 72%). 

The patients received vaccinations as either heterologous (combined) or homologous (single-type) doses of BNT162b2 and CoronaVac. The majority of the patients in both the HF and NHF groups received heterologous vaccinations (*n* = 43; 41.7% and *n* = 52; 50.3%, *p* = 0.002, respectively). Other relevant demographic data are presented in Table 1.

### 3.2. Anti-Spike Antibody Positivity

There was no significant difference in antibody positivity between patients with and without heart failure (OR: 1.5, 95% CI: 0.1–17.6, *p* = 0.725). However, a significant increase in antibody positivity was observed among patients with hyperlipidemia compared to those without (OR: 4.7, 95% CI: 1.6–13.5, *p* = 0.004) (refer to Table 2). Furthermore, there was no association found between antibody positivity and age (*p* = 0.158) and hypertension (*p* = 0.980) and diabetes (*p* = 0.751).

### 3.3. Anti-Spike Antibody Levels with Different Vaccines

A notable variance in antibody positivity emerged across different vaccine types. In contrast to CoronaVac alone, individuals receiving only BNT162b2 exhibited a significantly higher antibody positivity (OR: 11.0, 95% CI: 3.8–31.5, *p* < 0.001). Likewise, those who received a heterologous vaccination regimen involving both BNT162b2 and CoronaVac demonstrated a substantial increase in antibody positivity compared to CoronaVac alone (OR: 26.4, 95% CI: 8.8–79.1, *p* < 0.001) (Table 2).

### 3.4. Anti-Spike Antibody Levels with Different Time Intervals from the Last Vaccine Dose

As each day elapsed following the final vaccine dose, a noteworthy decline in anti-spike antibody positivity was evident, showcasing an odds ratio (OR) of 0.9 (95% CI: 0.9–0.9). The distinction in antibody positivity over time since the last vaccine dose was also assessed across the two groups. Notably, there was no statistically significant difference in the change in antibody positivity over time from the last vaccine dose between patients with and without heart failure (OR: 1.0, 95% CI: 0.9–1.0, *p* = 0.727).

**Figure 2:**
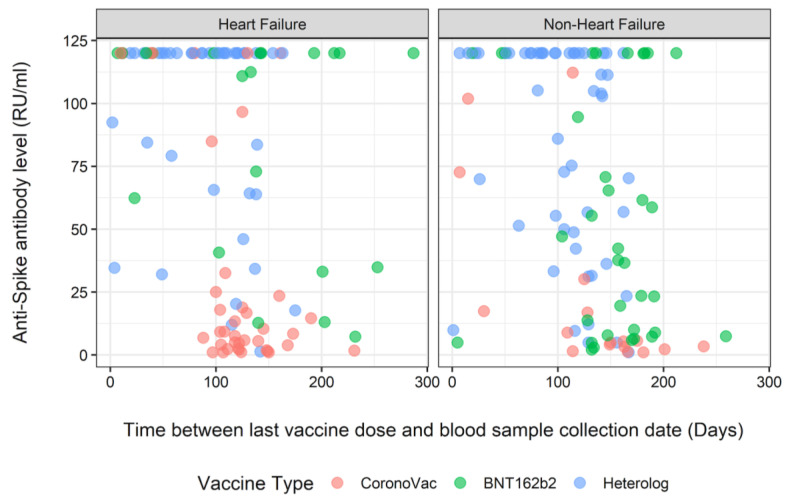
Distribution of anti-spike antibody levels over time according to vaccine type and heart failure status.

Figure 2 illustrates the anti-spike antibody levels in both the HF and NHF groups over the duration from the last vaccine dose to the blood sample collection date. Notably, among the patients vaccinated only with CoronaVac, antibody levels were approximately 120 RU/mL when the blood sample was taken in the initial days following the last vaccine dose. However, as time progressed to 100–200 days post-vaccination, the antibody levels dropped below 25 RU/mL.

**Figure 3:**
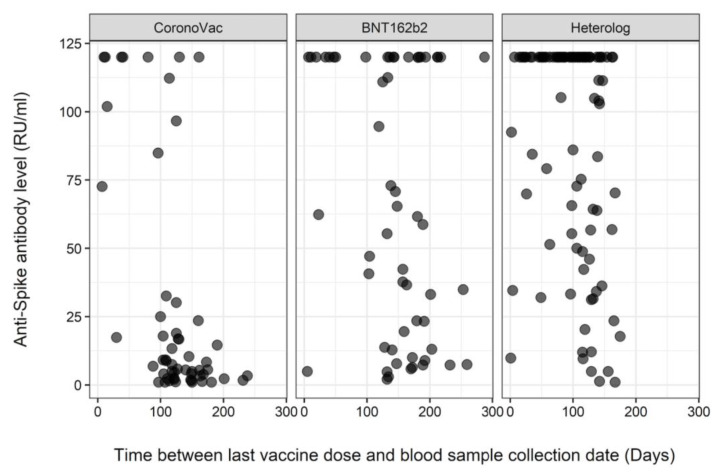
Distribution of anti-spike antibody levels for different vaccine types over time.

For the patients vaccinated with only with BNT162b2, a discernible pattern in the distribution of values was not apparent. However, it was noteworthy that even when the blood sample was taken after 200 days from the last vaccine dose, there were patients for whom the antibody levels were calculated at around 120 RU/mL (Figure 2 and Figure 3). There were no patients who received a heterologous vaccination and had blood samples taken 200 days after vaccination.

## 4. Discussion

The study findings revealed no significant differences in the overall anti-spike antibody positivity between patients with heart failure and those without it. A notable decline in anti-spike antibody positivity was observed with each passing day following the final vaccine dose, but this trend did not differ between the two groups. Compared with individuals receiving CoronaVac alone, individuals receiving only BNT162b2 or a heterologous vaccination demonstrated significantly higher antibody positivity. Between 100 and 200 days post-vaccination, recipients of CoronaVac alone exhibited a decrease in antibody levels below 25 RU/mL. Conversely, patients vaccinated with only BNT162b2 maintained antibody levels at around 120 RU/mL, even when the blood sample was collected after 200 days from the last vaccine dose. Additionally, there was a significant increase in antibody positivity among patients with hyperlipidemia compared to those without it.

SARS-CoV-2 infection has been a major health concern affecting a significant portion of the world’s population [11]. It has been more than three years since the pandemic became a global threat, and there is currently reasonable evidence that both the CoronaVac and BNT162b2 vaccines may be efficient in battling SARS-CoV-2 infection [2,4,12]. There are suggestions that comorbid and frail groups (such as HF patients) may be, to some extent, immunocompromised and may respond to vaccination less than expected by producing fewer anti-spike antibodies. However, there is no evidence for the comparative efficacy of the available vaccines in HF patients [13]. 

The synergistic effect of the humoral and cellular immune responses helps the host to fight against viral infections. Specific humoral immunity against SARS-CoV-2 in COVID-19 disease-infected patients and those with vaccinations has been described in a previous study [14]. Memory B cells play the central role in this humoral immune response. Goel et al. reported that mRNA vaccine-induced variant-specific memory B cells were maintained for at least six months [15]. Inactivated vaccines induce neutralizing antibodies after inoculation as well as cellular immune responses targeting SARS-CoV-2 proteins [16]. The immune response to SARS-CoV-2 vaccines may be impaired owing to immunity errors. Evidence suggests that in diseases like common variable immunodeficiency, anti-spike antibody production may be impaired, resulting in reduced humoral as well as cellular responses to SARS-CoV-2 vaccination [17,18,19].

It has previously been described that as a result of monocyte and T-cell activation, resulting from host/LVAD device interactions, LVAD recipients develop progressive defects in cellular immunity [6]. LVAD therapy may also induce defects in the humoral immune system, including a reduction in the number of CD4+ T cells, increased apoptosis of CD4+ and CD8+ T cells, and B-cell hyper-reactivity [20]. 

Both recent and historical data support the deposition of activated monocytes and T-cells on the LVAD surface, resulting in relative T-cell-directed immune suppression. Gene expression related to cellular immunity is regulated by seven days after the implantation of LVADs [6]. However, immune dysregulation begins before the device is implanted. Immune activation occurs in decompensated HF, with increased levels of the membrane attack complex as well as increased concentrations of interleukin-6, tumor necrosis factor-α, and interleukin-1 [5,6]. Itzhaki Ben Zadok et al. studied six months of immunogenicity to SARS-CoV-2 in 53 heart-transplant recipients and 18 LVAD patients who received two doses of BNT162b2. The authors observed higher seropositive rates in LVAD patients, which was attributed to the immunocompetent status and durability of the humoral immune response in this patient population [21]. In the present study, we observed no differences in anti-spike antibody positivity between the patients with and without heart failure. 

The durability of the antibody response post-COVID-19 vaccination across diverse populations has been previously explored [22]. Laing et al. reported detectable antibodies six months after SARS-CoV-2 vaccination. In the present study, the results revealed that a significant decline in antibody positivity was observed as time passed from the last vaccine dose; however, this decline did not differ between patients with and without heart failure. Additionally, we investigated the antibody-level variations associated with different vaccine types. As shown in Figure 2 and Figure 3, individuals who received only CoronaVac showed lower antibody levels 100–200 days post-vaccination compared to those who received only BNT162b2. Notably, within the BNT162b2 group, certain patients maintained antibody levels at around 120 RU/mL, even after 200 days from their last dose.

COVID-19 vaccines vary in their ability to elicit immune responses within the human body. There are studies available in the literature that compare the levels of anti-spike antibodies across various types of vaccines and in different patient populations [23]. A comparison between anti-spike antibody responses in individuals vaccinated with either CoronaVac or BNT162b2 was conducted by Mok et al. [1], who reported stronger humoral responses with BNT162b2 compared to CoronaVac. In line with these previous findings, the present study revealed that vaccination with BNT162b2 led to significantly higher antibody levels compared to vaccination with CoronaVac alone. 

The aging immune system undergoes changes favoring overall immune suppression, including both innate (neutrophils, monocytes, and macrophages) and adaptive (B- and T-cells) immunity. The increased utilization of these devices as a destination therapy, with 46% of LVADs being implanted in patients over 60 years old, often leads to age-related immune suppression at the time of device implantation and throughout the duration of the patient’s time with the device [24,25]. The dysregulation of immunity can also be observed with an increased inflammatory response in diabetes [26]. Tall et al., as suggested in their study, highlighted that hypercholesterolemia triggers the accumulation of cholesterol in macrophages and other immune cells, thereby promoting inflammatory responses. This involves the augmentation of toll-like receptor signaling, activation of inflammasomes, and an upsurge in the production of monocytes and neutrophils in the bone marrow and spleen [27]. In our present study, we investigated the impacts of age, hyperlipidemia, hypertension, and diabetes on anti-spike antibody positivity. Notably, hyperlipidemia was associated with increased antibody positivity (*p* = 0.004). This finding suggests a potential link to an activated immune response induced by hyperlipidemia, resulting in an elevation in antibody positivity. Nevertheless, there was no association found between increased antibody positivity and age (*p* = 0.158), hypertension (*p* = 0.980), and diabetes (*p* = 0.751).

## 5. Study Limitations

This study has several limitations. The assessment of antibody levels was affected by variations in vaccine types and the duration since the last vaccine dose, which prevented independence from these influential factors. This limited our ability to make definitive statements about the changes in antibody levels over time, highlighting the need for a longitudinally collected dataset. These factors could impact and limit the generalizability of the results. Furthermore, the single-center approach introduces potential biases and may not fully capture the characteristics of the broader population, thereby affecting the external validity of the study’s findings.

## 6. Conclusions

This study revealed a lack of advantage in anti-spike antibody positivity post-vaccination against SARS-CoV-2 for both cohorts, irrespective of the presence of heart failure. Although there was an observable wane in anti-spike antibody positivity over time following the last vaccine dose, this decline exhibited uniformity across both groups. Although both cohorts showed similar levels of antibody positivity, a noteworthy pattern emerged in the increased positivity of antibody levels associated with BNT162b2 vaccination. This finding, even after a considerable duration since the final vaccine dose, implies the vaccine’s heightened efficacy in fortifying protection against infection. The sustained and elevated antibody response further accentuates the distinct advantage conferred by BNT162b2, which emphasizes its role as a robust defender against infection.

## Figures and Tables

**Table 1:** Baseline characteristics.

Variable	Heart Failure (*n* = 103)	Non-Heart Failure (*n* = 104)	*p* Value
**Age,** years, median (Q1–Q3)	56.0 (48.0–62.5)	63.0 (56.0–70.2)	<0.001 ^1^
**Hypertension**	40 (38.8%)	64 (61.5%)	0.001 ^2^
**Diabetes**	22 (21.3%)	43 (41.3%)	0.002 ^2^
**Hyperlipidemia**	15 (14.5%)	47 (45.1%)	<0.001 ^2^
**Gender**			0.840 ^2^
Female	25 (24.3%)	24 (23.1%)	
Male	78 (75.7%)	80 (76.9%)	
**Vaccine**			0.002 ^2^
BNT162b2	21 (20.8%)	35 (33.6%)	
CoronaVac	39 (37.8%)	17 (16.3%)	
Heterologous (CoronaVac and BNT162b2)	43 (41.7%)	52 (50.3%)	
Period *, Days, Median (Q1–Q3)	118.0 (83.5–140.0)	130.5 (97.8–162.0)	0.040 ^1^
**Procedure**			
Isolated CABG		65 (62.5%)	
Single MVR		4 (3.8%)	
Single AVR		10 (9.6%)	
Single TVR		1 (0.9%)	
ASD Repair		1 (0.9%)	
Septal Myectomy		1 (0.9%)	
Bentall Procedure		3 (2.8%)	
CABG with AVR		4 (3.8%)	
CABG with MVR		1 (0.9%)	
CABG with CEA		2 (1.9%)	
Double-Valve Operation		8 (7.6%)	
Triple-Valve Operation		1 (0.9%)	
CABG with Ascending Aorta Replacement		2 (1.9%)	
**Etiology of Heart Failure**			
ICMP	48 (46.6%)		
DCMP	50 (48.5%)		
RCMP	2 (1.9%)		
HCMP	4 (3.8%)		
**Ventricular Assist Device**			
Abbott HeartMate 2	5 (5.8%)		
Abbott HeartMate 3	19 (22%)		
Medtronic HeartWare HVAD	62 (72%)		

*p* values are determined by ^1^ Mann–Whitney U and ^2^ Pearson’s Chi-Squared tests. CABG = coronary artery bypass grafting; MVR = mitral valve replacement; AVR = aortic valve replacement; ASD = atrial septal defect; CEA = carotid endarterectomy; ICMP = ischemic cardiomyopathy; DCMP = dilated cardiomyopathy; RCMP = restrictive cardiomyopathy; HCMP = hypertrophic cardiomyopathy. *, Period: The duration between the last vaccination dose and the collection of the blood sample.

**Table 2:** Analysis of anti-spike antibody positivity in heart failure and non-heart failure groups, accounting for potential confounding factors.

	B	S.E.	*p* Value	OR	95% C.I.	
					Lower	Upper
**Model 1**						
**Hyperlipidemia ** (Reference Category: No)	1.550	0.540	0.004	4.711	1.635	13.575
Period *	−0.021	0.006	0.001	0.979	0.967	0.991
**Vaccine Group** (Reference Category: CoronaVac)						
BNT162b2	2.403	0.535	<0.001	11.059	3.877	31.549
Heterologous (CoronaVac and BNT162b2)	3.274	0.560	<0.001	26.412	8.810	79.189
Model 2 **						
**Heart Failure** (Reference Category: Non-Heart Failure)	0.437	1.240	0.725	1.548	0.136	17.602
Heart Failure * Period (Reference Category: Non-Heart Failure)	0.003	0.008	0.727	1.003	0.987	1.019

* Period: The duration between the last vaccination dose and the collection of the blood sample; S.E.: standard error; OR: odds ratio; C.I.: confidence interval. ** Model 2 also included hyperlipidemia, period, and vaccine groups. Model 1: omnibus tests of model: $\chi^{2}(4)=73.55$ and *p* < 0.001; Hosmer–Lemeshow test: $\chi^{2}(8)=11.07$ and *p* = 0.198; Nagelkerke R^2^ = 0.445. Model 2: omnibus tests of model: $\chi^{2}(6)=76.70$ and *p* < 0.001; Hosmer–Lemeshow test: $\chi^{2}(8)=10.57$ and *p* = 0.227; Nagelkerke R^2^ = 0.460.

## Data Availability

The data are available within the article.
